# Serine protease SP105 activates prophenoloxidase in Asian corn borer melanization, and is regulated by serpin-3

**DOI:** 10.1038/srep45256

**Published:** 2017-03-30

**Authors:** Yuan Chu, Fang Hong, Qizhi Liu, Chunju An

**Affiliations:** 1 Department of Entomology, China Agricultural University, Beijing, 100193, China

## Abstract

Melanization reaction, resulting from the activation of prophenoloxidase, is a vital immune response in insects for encapsulating and killing the invasive organisms. Prophenoloxidase needs to be proteolytically activated by its upstream prophenoloxidase-activating protease (PAP) in melanization. Identification and characterization of PAPs facilitates the understanding of the molecular mechanisms involved in insect immunity. We here cloned a full-length cDNA for a serine protease, named as SP105, from Asian corn borer, *Ostrinia furnacalis* (Guenée). The open reading frame of *SP105* encodes 424-amino acid residue protein with a 19-residue signal peptide. Sequence comparison indicates that SP105 is most similar to *Manduca sexta* PAP3, a defined prophenoloxidase-activating protease. qRT-PCR analysis showed that *SP105* mRNA levels increased significantly after a bacterial injection. Recombinant SP105 directly cleaved and activated Asian corn borer prophenoloxidase and therefore acted as the prophenoloxidase-activating protease. Additionally, SP105 formed SDS-stable complexes with a serine protease inhibitor, serpin-3, and its activity in activating prophenoloxidase was efficiently inhibited by serpin-3. Our work thus illustrated a prophenoloxidase-activating protease and revealed its regulation by serpin-3. The results would allow further advances in the understanding of the melanization in Asian corn borer and other insects.

Most insects lack a typical adaptive immune system and mainly rely on the innate immune response for defense against the infection of pathogens or parasites^1^,^2^,^3^. Insect innate immune response has striking similarities to mammalian innate immune response, and also consists of humoral and cellular responses^4^. Melanization reaction is a prominent humoral response in insects, and combines with other immune responses such as antimicrobial peptide production, phagocytosis, nodulation and encapsulation to kill and eliminate the invading microorganisms or parasites^5^,^6^,^7^.

Current understanding of the mechanism of melanization is mainly from powerful genetic studies in fruit fly, *Drosophila melanogaster*^1^,^5^,^6^,^7^,^8^,^9^, and biochemical studies in relatively large insects, such as the silkworm, *Bombyx mori*^10^,^11^, the tobacco hornworm, *Manduca sexta*^12^,^13^,^14^, and the beetle *Tenebrio molitor*^15^,^16^. During insect melanization reaction, soluble pattern-recognition proteins initially recognize non-self molecular patterns from the invading microorganisms^17^,^18^. This recognition triggers the sequential activation of a series of serine proteases, culminating in the activation of prophenoloxidase-activating protease (PAP), also known as prophenoloxidase-activating enzyme or factor (PPAE or PPAF)^13^,^16^,^19^. Activated PAP converts inactive prophenoloxidase (PPO) to phenoloxidase (PO)^12^,^15^,^20^,^21^. Ultimately, active phenoloxidase catalyzes the oxidation of phenols to quinones, which spontaneously polymerize to form melanin^22^. Therefore, identification and characterization of PAPs helps to better understand the molecular mechanisms involved in the melaniztaion. Several PAPs have been demonstrated in only limited insect species including CLIPB9 in *Anopheles gambiae*^20^, PPAE in *B. mori*^23^, MP2 (CG3066) in *D. melanogaster*^9^,^24^, PPAF-1 in *Holotrichia diomphalia*^25^, PAP-1/-2/-3 in *M. sexta*^12^,^26^, and SPE in *T. molitor*^16^,^19^. In other insects, knowledge about PAP is still absent.

Serine proteases, especially those with one or two clip domain(s), are actively involved in prophenoloxidase-activation cascade^3^,^6^. Clip domain serine protease consists of the clip domain at the N-terminus terminus and a catalytic domain at the C-terminus^27^,^28^. They are secreted into hemolymph as inactive precursors and require the specific proteolytic cleavage at the activation site for conducting functions^3^,^27^. This activation is often regulated by members of the serine protease inhibitor (serpin) superfamily^29^,^30^,^31^. Upon binding to the target protease, serpin is cleaved at the scissile bond in the reactive center loop by its target protease and subsequently undergoes a large conformation change. Ultimately, serpin becomes covalently linked to the target protease, which is therefore irreversibly inhibited^30^,^32^. Serpin has been reported to participate in the regulation of insect melanization, such as *Aedes agypti* SRPN1 and SRPN2^21^, *A. gambiae* SRPN2 and SRPN6^20^,^33^, *D. melanogaster* Spn27A, Spn28D, Spn77Ba, and Spn5^34^,^35^,^36^,^37^, *M. sexta* serpin-1, -3 through -5, and -7^26^,^38^,^39^,^40^, and *T. molitor* SPN40, SPN55 and SPN48^41^. However, the cognate protease the serpin inhibits has not been clearly revealed in most insects.

The Asian corn borer, *Ostrinia furnacalis* (Guenée), is an important insect pest in Asia, causing serious damage on corn, sorghum, millet and other crops^42^. The molecular and biochemical mechanisms involved in Asian corn borer against pathogen infection are largely unknown, possibly due to the unavailability of genomic information. In our previous work, we have characterized the transcriptome of Asian corn borer larvae^43^, and indicated that two serine proteases, SP1 and SP13 mediated the melanization response^44^. In this study, we cloned a full-length cDNA for a serine protease, named as *SP105* (GenBank: KT751521) from Asian corn borer. SP105 transcript was elevated dramatically upon bacterial challenge. Recombinant SP105 directly cleaved and activated Asian corn borer prophenoloxidase. And the activity of SP105 in cleaving prophenoloxidase was regulated by a defined serine protease inhibitor, serpin-3.

## Results

### Molecular cloning and sequence analysis of Asian corn borer *SP8* and *SP105*

**Figure 1:**
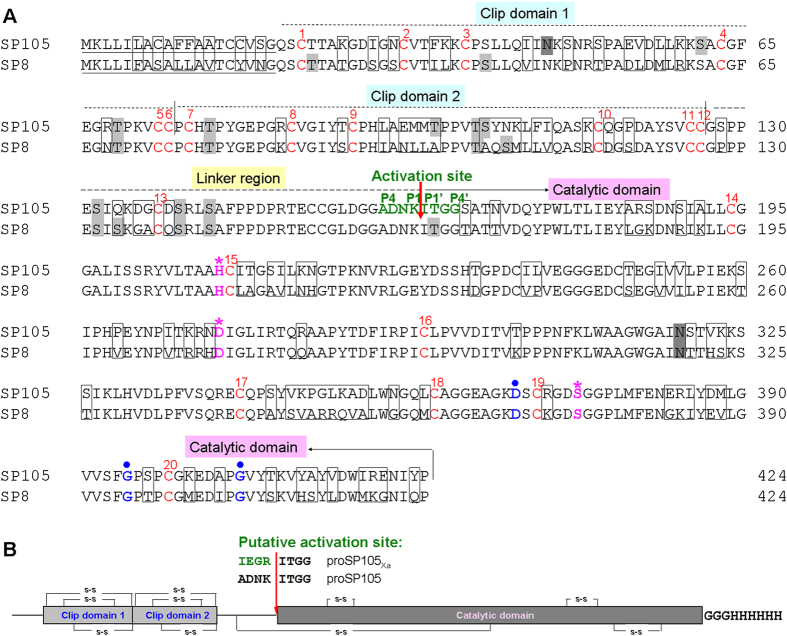
Sequence analysis of Asian corn borer *SP8* and *SP105*. (**A**) Sequence comparison between SP8 and SP105. The different amino acids in two deduced protein sequences are *boxed*. The predicted secretion signal peptide is *underlined*. Putative *N*-linker and *O*-linked glycosylation sites are heavily and lightly shaded, respectively. The overall domain division (two clip domains and one catalytic domain) are indicated above the sequences. The absolutely conserved Cys residues in clip domain serine proteases are numbered and shown in *red*. They are predicted to form ten disulfide bonds (1–5, 2–4, 3–6; 7–11, 8–10, 9–12; 13–16, 14–15, 17–18, 19–20) based on the determined structure in *M. sexta* PAP2^57^ and *H. diomphalia* PPAF-I^58^. The residues of the catalytic triad (His, Asp, Ser) are shown in *purple* and indicated by *asterisks*. The important determinants of the specificity pocket in the catalytic domain are shown in *blue* and maked by *circles*. The potential cleavage activation sites are shown in *green* and labeled by *red arrow*. (**B**) Schematic representation of recombinant SP105. The disulfide linkage in (**A**) are shown in *lines with a symbol (s*-*s*). The position of the peptide bond cleaved during activation is indicated by *red arrow*. In proSP105_Xa_, the putative activation site was changed from ADNK to IEGR. Three glycine and six histidine residues were added to the carboxyl terminus of SP105 and shown in *bold*.

We have identified 13 potential clip domain serine proteases from Asian corn borer larvae^43^. Among them, serine protease 8 (SP8) was predicted to mediate the menalization response in Asian corn borer^44^. As a first step to characterize SP8’s function, we designed primers on the basis of the known transcriptome^43^ and tried to clone *SP8* in full length nucleotide sequence. Interestingly, we also identified another serine protease gene, nominated as *SP105*, in addition to the expected *SP8*. The cDNA sequences of *SP8* and *SP105* were successfully submitted to NCBI, with GenBank accession number as KT751522 and KT751521, respectively. They shared 82.4% identity in nucleotide acid sequences. The conceptual protein deduced from nucleotide sequence of *SP8* and *SP105* consists of 424 amino acid residues, including a predicted 19-residue secretion signal peptide. There are one and two putative *N*-linked glycosylation sites, and eleven and eight potentially *O*-linked glycosylation sites in deduced SP8 and SP105, respectively (Fig. 1A). The calculated molecular mass and isoelectric point of the mature protein are 43.1 kDa and 6.38 for SP8, 43.6 kDa and 6.35 for SP105, respectively.

Asian corn borer SP8 and SP105 are each composed of two amino-terminal clip domains connected by a linker region to a carboxyl-terminal S1 family serine protease domain containing a catalytic triad consisting of His, Asp, and Ser residues (Fig. 1). They are most similar in amino acid sequence to *M. sexta* PAP3, a clip domain serine protease directly activating prophenoloxidase in melanization process^13^ (Fig. S1). The predicted proteolytic activation sites (↓) are located at ADNK^163^↓ITGG^167^ in both SP8 and SP105 (Fig. 1B). The important determinants of the enzyme specificity are predicted to be Asp^368^, Gly^395^, and Gly^406^ in SP8 and SP105 (Fig. 1A), suggesting they are trypsin-like protease cleaving its substrate after arginine or lysine residue^45^.

As mentioned above, we successfully obtained two full-length cDNA sequences during amplifying Asian corn borer *SP8*. However, three clones in nine sequenced *SP8* samples had a mutant residue from Cys^38^ to Arg^38^, which is absolutely conserved in all defined clip domains. Additionally, recombinant SP8 with Arg^38^ failed to be activated with unknown reasons (Fig. S2). Therefore, we only focused on *SP105* in the studies that followed.

### Gene expression profiles of Asian corn borer *SP105*

**Figure 2:**
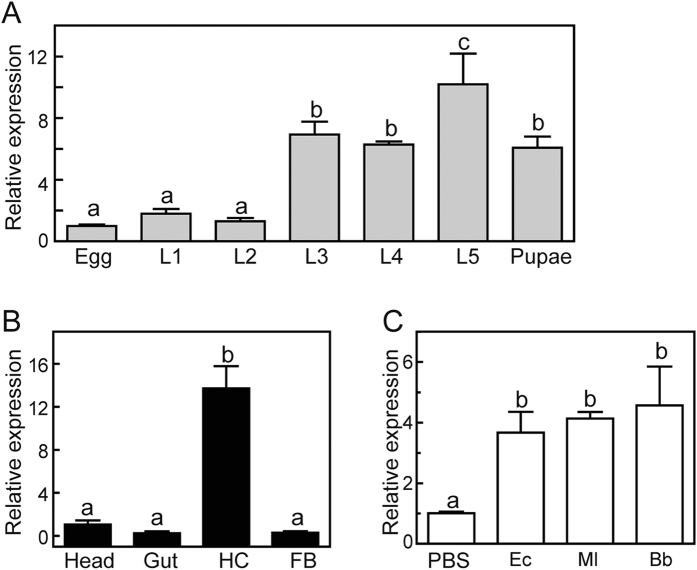
qRT-PCR analysis of *SP105* expression in Asian corn borer. (**A**) Expression profiles of SP105 in Asian corn borer at different stages of development. RNA was extracted from the whole bodies collected from eggs, first-instar (*L1*), second-instar (*L2*), third-instar (*L3*), fourth-instar (*L4*), fifth-instar (*L5*) larvae, and pupae. The *rpL8* was used as an internal control. (**B**) Expression patterns of *SP105* in different tissues of Asian corn borer larvae. Tissues including head, gut, hemocytes (*HC*), and fat bodies (*FB*) were collected from day 0, fifith instar larvae for RNA extraction. qRT-PCR was performed to assess the transcript level of *SP105*. The *rpL8* was used to normalize the templates. (**C**) Expression profiles of *SP105* in Asian corn borer larvae upon microbial challenge. Day 1, fifth instar larvae were infected with water, *E. coli (Ec*), *M. luteus (Ml*), or *B. bassiana (Bb*). RNA was prepared from the whole bodies 20 h after injection. qRT-PCR was used to analyze the transcript change of SP105 with *rpL8* as an internal standard to indicate a consistent total mRNA amount. The bars represent mean ± S.D. (*n* = 3). Bars labeled with different letters are significantly different (one-way ANOVA followed by Newman-Keuls test, *P* < 0.05).

We analyzed the mRNA levels of Asian corn borer *SP105* in the various development stages, different tissues, or different pathogen inducements using qRT-PCR methods. *SP105* transcripts in fifth instar larvae were significantly more than that in other developmental stages. Although *SP105* expression level remained consistent in three, fourth instar larvae and pupae, it was still significantly higher than in the egg, first and second instar larval stage (Fig. 2A). In different tissues, *SP105* was expressed at significantly higher levels in hemocytes than in head, gut, and fat bodies. *SP105* transcripts increased up to 14 folds in hemocytes (Fig. 2B). Moreover, qRT-PCR assay showed that *SP105* mRNA levels increased significantly in the larva challenged by *Escherichia coli, Micrococcus luteus* or *Beauveria bassiana* conidia (Fig. 2C).

### Purification and activation of recombinant proSP105_Xa_

**Figure 3:**
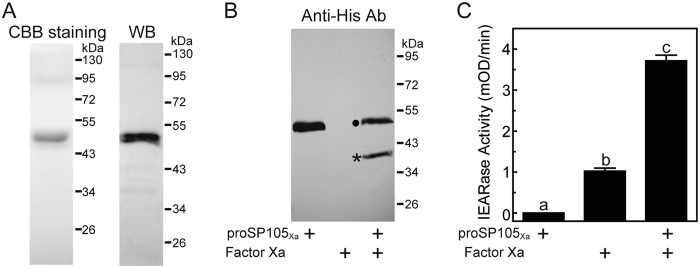
Purification and activation assay of recombinant proSP105_Xa_. (**A**) SDS-PAGE and immunoblot analysis of purified proSP105_Xa_. The purified proSP105_Xa_ (0.25 μg) was treated with SDS sample buffer containing DTT and separated by 10% SDS-PAGE followed by coomassie brilliant blue staining (*left panel*) or immunoblotting with anti-His as primary antibodies (*right panel*). The sizes and positions of the molecular weight standards are indicated on the *right*. (**B**) Detection of the activation of purified proSP105_Xa_ by Factor Xa by Western blot. After incubation of the purified recombinant proSP105_Xa_ (0.25 μg) with Factor Xa (0.2 μg), the mixtures were subjected to 10% SDS-PAGE for immunoblot analysis using Anti-His antiserum. Circle, proSP105_Xa_; asterisk, catalytic domain of proSP105_Xa_. (**C**) Detection of the activation of purified proSP105_Xa_ by Factor Xa by spectrophotometric assay using IEAR*p*NA as a substrate. The bars represent mean ± S.D. (*n* = 3). Bars labeled with different letters are significantly different (one-way ANOVA followed by Newman-Keuls test, *P* < 0.05).

In order to investigate the potential function of SP105 in Asian corn borer, we produced active SP105 *in vitro*. Considering SP105 is expressed as a zymogen (proSP105) and its endogenous activating enzyme is currently unknown, we produced a recombinant form of proSP105 (proSP105_Xa_) in Sf9 cells. In proSP105_Xa_, the predicted activation site was manually mutated from ADNK^163^ to IEGR^163^ to permit its activation by commercially available bovine Factor Xa. Recombinant proSP105_Xa_ was secreted into medium using its own secretion signal peptide. SDS-PAGE analysis indicated that purified proSP105_Xa_ had an apparent mass of ~50 kDa, approximately 7 kDa larger than that predicated based on its cDNA sequence (Fig. 3A). The increased mass is likely due to glycosylation, as the protein sequence contain ten putative glycosylation sites (Fig. 1A).

Incubation of purified proSP105_Xa_ with Factor Xa resulted in decreased intensity of the 50-kDa zymogen band and the appearance of a 38-kDa band corresponding to the catalytic domain of proSP105_Xa_ (Fig. 3B), as expected for activation cleavage. After conditioned medium containing recombinant wild type proSP105 with 6×His tag was incubated with Asian corn borer plasma, the new band representing the catalytic domain of proSP105 appeared at the same position (38 kDa) (Fig. S3). It suggests that the cleavage of proSP105_Xa_ by Factor Xa could simulate the activation of proSP105 *in vivo*. Additionally, the activation of proSP105_Xa_ by Factor Xa was confirmed by testing whether activated SP105_Xa_ could hydrolyze the colorimetric substrate IEAR*p*NA. As shown in Fig. 3C, proSP105_Xa_ lacked IEARase activity, but after the zymogen was activated by Factor Xa, IEARase activity increased significantly above that of Factor Xa alone, which could also hydrolyze the substrate. These results indicated that Factor Xa cleaved and activated proSP105_Xa_.

### SP105_Xa_ activated native and recombinant Asian corn borer PPO

When purified proSP105_Xa_ zymogen was added into Asian corn borer plasma, the 38-kDa band corresponding to the catalytic domain was detected (Fig. S4A), although the putative activation sites in proSP105_Xa_ had been mutated from original ADNK^163^ to IEGR^163^. The addition of recombinant proSP1_Xa_, another Asian corn borer serine protease with IEGR^114^ as activation sites^44^, also led to the appearance of 33-kDa product corresponding to the catalytic domain of proSP1_Xa_ (Fig. S4B). It seems that unknown protease in Asian corn borer plasma possessed partial activity of Factor Xa to cleave its downstream protease after IEGR residues, and therefore could weakly activate proSP105_Xa_.

**Figure 4:**
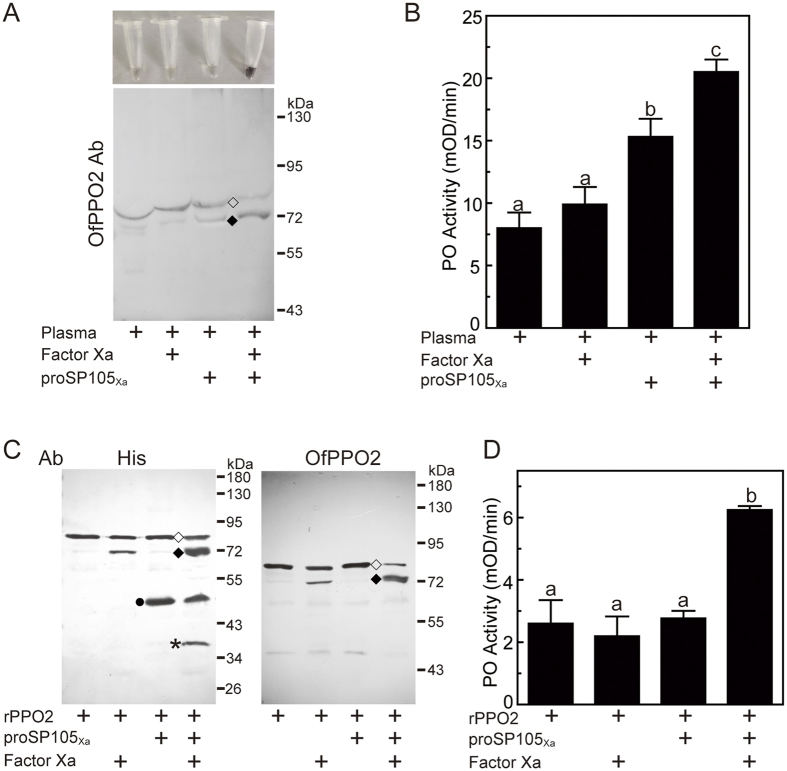
Proteolytic activation of Asian corn borer PPO2 by Factor Xa-activated SP105. Factor Xa-activated SP105_Xa_ cleaved Asian corn borer PPO2 in plasma (**A**) and as purified protein (**C**), causing a significant increase in PO activity (**B**,**D**). In (**A**,**C**), the sizes and positions of the molecular weight markers are indicated on the *right*. His or Asian corn borer PPO2 antiserum was used as primary antibodies in immunoblot analysis. Circles, proSP105_Xa_; asterisks, catalytic domain of proSP105_Xa_; hollow diamonds, PPO2 zymogen; solid diamonds, cleaved and activated PO2. In (**B**,**D**), the bars represent mean ± S.D. (*n* = 6). Statistically significant differences are represented by different letters (one-way ANOVA followed by Newman-Keuls test, *P* < 0.05).

After Asian corn borer plasma was incubated with Factor Xa alone, the antibodies against Asian corn borer PPO2 identified an 80-kDa band corresponding to PPO2 zymogen. The incubation of Asian corn borer plasma with proSP105_Xa_ zymogen resulted in the appearance of a new band with low intensity at 72-kDa corresponding to Asian corn borer PO2, and a slight increase in PO activity (Fig. 4A,B). After plasma was incubated with Factor Xa-activated proSP105_Xa_, the 72-kDa band corresponding to Asian corn borer PO2 was observed at higher intensity, the reaction mixture turned black, and PO activity increased significantly (Fig. 4A,B). It indicates that active SP105_Xa_ causes cleavage and activation of Asian corn borer PPO2.

To explore whether PPO is a substrate of SP105, we incubated SP105_Xa_ and recombinant Asian corn borer PPO2. As in Asian corn borer plasma, western blot analysis of purified PPO2 detected a band with apparent molecular weight of 80 kDa. No change was observed after incubation with proSP105_Xa_ zymogen. The addition of Factor Xa alone into plasma led to the weak appearance of 72-kDa band corresponding to Asian corn borer PO2, but no significant change in PO activity, suggesting the weak cleavage of recombinant PPO2 by Factor Xa. However, when active SP105_Xa_ was incubated with purified PPO2, the band of 72 kDa was observed with strong intensity and PO activity of the mixture increased significantly (Fig. 4C,D).

### SP105_Xa_ was directly inhibited by Asian corn borer serpin-3

**Figure 5:**
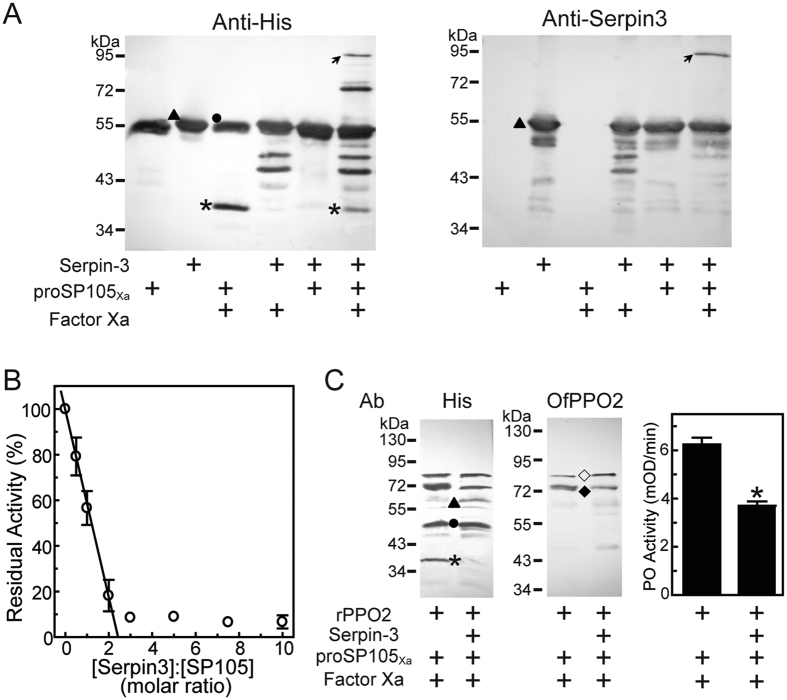
Inhibition assay of SP105 by Asian corn borer serpin-3. (**A**) SDS-stable complex formation between SP105 and serpin-3. ProSP105_Xa_ (0.2 μg) was activated by 0.2 μg of Factor Xa, and then incubated with 0.17 μg of purified serpin-3 at room temperature. After 30 min, the reaction mixtures were subjected to 10% SDS-PAGE and immunoblot analysis using antiserum against His (*left panel*) or Asian corn borer serpin-3 (*right panel*). Size and positions of molecular mass standards are indicated to the *left* of each blot. Circles, proSP105_Xa_; asterisks, catalytic domain of proSP105_Xa_; triangles, serpin-3; arrows, serpin-3/SP105_Xa_ complex. (**B**) Stoichiometry for inhibition of SP105 by serpin-3. Purified recombinant serpin-3 was incubated with Factor Xa-activated SP105_Xa_ at various molar ratios for 30 min at room temperature. The residual IEARase activities of SP105_Xa_ was plotted as mean ± S.D. (*n* = 3) against the corresponding molar ratios of serpin-3 and SP105_Xa_. (**C**) Serpin-3 inhibited the cleavage of recombinant PPO2 by SP105. Factor Xa-activated SP105_Xa_ (0.2 μg) was incubated with a 1-fold molar excess of serpin-3, then incubated with recombinant Asian corn borer PPO2 (0.3 μg). The mixtures were subjected to 7.5% SDS-PAGE and immunoblotting using His (*left panel*) or PPO2 (*middle panel*) antiserum. Circle, proSP105_Xa_; asterisk, catalytic domain of proSP105_Xa_; triangle, serpin-3; hollow diamond, PPO2 zymogen; solid diamond, activated PO2. PO activity of the mixtures (mean ± S.D., *n* = 4) was monitored using dopamine as substrate (*right panel*). Asterisk means that is significantly different from the control (unpaired *t* test, two-tailed *p* < 0.05).

In a previous study, we identified a serine protease inhibitor, serpin-3, which regulates melanization cascade in Asian corn borer^46^. To investigate whether serpin-3 functioned via inhibiting SP105, we firstly checked whether serpin-3 could form an SDS-stable complex with SP105_Xa_ because the formation of such complex is a characteristic feature of serpin-target protease reaction^30^. We mixed SP105_Xa_ with recombinant serpin-3, and detected the appearance of a high molecular weight complex by western blot, using antibodies against His or serpin-3. Anti-His serum recognized both proSP105_Xa_ zymogen and serpin-3 as ~53-kDa band, which migrated to the same apparent position in SDS-PAGE (Fig. 5A, left panel). The incubation of recombinant serpin-3 with Factor Xa alone resulted in the appearance of two bands at ~48 and 45 kDa, possibly due to the nonspecific degradation of serpin-3 by Factor Xa. However, when serpin-3 was mixed with Factor Xa-activated SP105_Xa_, the intensity of the 38-kDa band corresponding to the catalytic domain of proSP105_Xa_ decreased, and a new immunoreactive band at ~92-kDa position (the expected size of a serpin-3/SP105 complex) was observed, which was also recognized by antibody against serpin-3 (Fig. 5A, right panel).

To confirm that this complex formation indeed leads to the inhibition of SP105, we tested the IEARase activity of SP105_Xa_ in the presence of serpin-3. SP105_Xa_’s activity decreased linearly as serpin-3 concentration increased (Fig. 5B). The stoichiometry of inhibition was 2.4, indicating that under the experimental conditions serpin-3 almost exclusively acted as an inhibitor rather than a substrate of SP105.

Since SP105 could cleave Asian corn borer PPO2, inhibition of SP105 by serpin-3 theoretically would suppress its cleavage of the substrate PPO2. To test this hypothesis, we incubated Asian corn borer PPO2 with Factor Xa-activated SP105_Xa_ in the absence or presence of serpin-3. When SP105 was pre-treated with serpin-3, more PPO2 was detected as 80-kDa band corresponding to PPO2 zymogen, and the 72-kDa band representing active PO2 decreased in intensity (Fig. 5C). The PO activity of the reaction mixture containing serpin-3 decreased significantly (Fig. 5C, right panel). Taken together, SP105 was inhibited by serpin-3.

## Discussion

Melanization of invading microbes is one important strategy that protects insects against infection^1^,^2^,^3^. In melanization cascade, prophenoloxidase (PPO)-activating protease (PAP) functions as the terminal protease to convert PPO into active phenoloxidase (PO)^12^,^13^,^15^,^20^,^21^. Although PAPs have been investigated for many years, understanding of PAPs in most insects is still incomplete. Here, we identified a serine protease gene, *SP105*, from Asian corn borer during amplifying expected *SP8*. Recombinant SP105 protein directly cleaved and activated Asian corn borer PPO2 *in vivo* and *in vitro*, which suggests that SP105 acts as a PAP in Asian corn borer melanization. In addition, we clarified that SP105’s activity was regulated by a serine protease inhibitor, serpin-3.

So far, veritable PAPs have only been determined in some model insects such as *A. gambiae*^20^, *B. mori*^23^, *D. melanogaster*^9^,^24^, *H. diomphalia*^25^, *M. sexta*^12^,^26^, and *T. molitor*^16^,^19^ (Fig. S1). In our previous work, we revealed that Asian corn borer SP13 directly cleaved *D. melanogaster* PPO1, and therefore potentially functioned as a PAP in Asian corn borer^44^. In this study, we resolved the previous problem and successfully obtained soluble Asian corn borer endogenous PPO, which meant we could perform experiments to check whether SP105 enable to cleave and activate its conspecific PPO. The results indicated that SP105 indeed activated native and recombinant Asian corn borer PPO2 (Fig. 4), and works as a PAP in PPO activation pathway. Therefore, we have identified two PAPs in Asian corn borer larvae so far. Similar situation also existed in *M. sexta*, in which three PAPs (PAP-1/-2/-3) have been characterized^12^,^26^. However, only one PAP was reported in other insects except for these two insect species. Based on the findings from Asian corn borer and *M. sexta*, we postulate that there should be more PAPs to be identified than currently illustrated in most insects. It ought to be common that multiple PAPs cooperatively activate PPOs in the melanization reactions, considering the key roles of PO in the melanization and the importance of the melanization against the microbial infection in insects.

In addition, it is notable that Asian corn borer SP105 also cleaved *D. melanogaster* PPO (Fig. S5). Consistently, Asian corn borer SP13 activated native and recombinant *D. melanogaster* PPO^44^, and *A. gambiae* CLIBP9 activated *M. sexta* PPO^20^. It suggests that the cleavage of PPO by PAP is not completely restricted in the conspecific insects. The possible reason is that PPOs from heterogeneous insects have the identical or highly conserved putative activation cleavage sites^33^,^46^, and its upstream activating protease recognizes and cleaves the same cleavage site without regard for the resource of PPO substrate. Therefore, in some small-size insects with limited amount of hemolymph for PPO purification or in the insects lacking sequence information for recombinant PPO production, it might be a reasonable strategy to make use of available heterologous PPO to clarify the putative function of a serine protease in PPO activation. Furthermore, some reports indicated that the proteases secreted from the invading microorganisms, such as fungal virulence factor Pr1, could proteolytically activate Persephone (Psh) protease in *D. melanogaster* Toll pathway^47^,^48^. It will be interesting to explore whether the secreted protease(s) from invading microbes also could directly activate host PPO since PAP cleaving PPO is not necessarily specific.

Although SP13 and SP105 redundantly function as PAP in Asian corn borer melanization response, there is still some remarkable differences between them. Firstly, SP13 and SP105 are typical clip-domain serine proteases containing type-2 clip domain with two helices between Cys-3 and Cys-4^27^,^28^,^43^ (Fig. 1). Clip domains are proposed to be sites for interactions of proteases with their activators, cofactors, and substrates^3^,^27^. SP13, most similar in amino acid sequences to *M. sexta* PAP1, contains a single type-2 clip domain, whereas SP105 which has the highest similarity to *M. sexta* PAP3 contains two type-2 clip domains (Fig. S1). Secondly, SP13 zymogen was activated by SP1 in PPO activation cascade in Asian corn borer^44^. However, SP1 failed to cleave proSP105 in our reduplicative experiments (Fig. S6), suggesting that SP1 acts upstream of proSP13, but not of proSP105, in Asian corn borer melanization. Similar results have been also reported in *M. sexta* PPO activation response, in which proPAP1 and proPAP3 are involved in two branched pathways and is activated by two separate serine protease, HP6 and HP21, respectively^13^,^14^. Therefore, we conclude that SP105 and SP13 belong to different type of PAPs and propose a hypothesis for the PPO activation in Asian corn borer. More than one cascade contributes to the PPO activation in Asian corn borer upon the challenge of foreign microbes. In one branch, the microbial infection leads to the activation of unknown serine protease(s) which then activates proSP1. Active SP1 then processes proSP13, and active SP13 in turn activates PPO. In another branch, the invading of pathogens or parasites results in the sequential activation of a serine protease other than SP1, then proSP105 is proteolytically activated. Active SP105 further converts PPO to PO. Other cofactors such as serine protease homologs (SPHs) also participate in this process. SPHs are similar in amino acid sequence to S1 family serine proteases but lack proteolytic activity due to the mutation of the catalytic residues^49^. SPHs have been reported to be essential for the prophenoloxidase activation in *M. sexta*^50^ and *H. diomphalia*^51^. We infer that there also exist similar SPH(s) in Asian corn borer to facilitate SP105 in activating PPO.

On the other hand, the activity of SP105 in cleaving PPO was blocked by serpin-3 (Fig. 5). In our previous report, serpin-3 also prevented SP13 from cleaving PPO^46^. It is not surprising that a single serpin regulates multiple target proteases. Actually, *M. sexta* serpin-3 inhibits all three PAPs in PPO activation cascade^38^,^40^. Serpin-1J in *M. sexta* not only regulates PAP-1 to -3 in melanization, but also restricts HP8 in Toll pathway^26^,^40^,^52^. Target specificity of inhibitory serpins is controlled by the sequence and tertiary structure of their reactive center loop, which allows the binding and cleavage by target proteases^32^,^33^. Serpin is thus kind of substrate of its target protease to some extent. We inferred that the serine proteases with the common downstream substrate would be potentially inhibited by the same serpin in insect immune response. More evidence will be presented to support this hypothesis as more serpin - target protease regulatory units are characterized in other insects. It also provides a useful clue to seek more cognate proteases for a specific serpin, or to identify the putative serpin for a serine protease which has identical substrate preference to the known protease in a defined serpin-protease unit.

## Methods

### Molecular cloning and sequence analysis of Asian corn borer *SP8* and *SP105*

In our previous study, a clip domain serine protease gene, *SP8*, was predicted to mediate the melanization response in Asian corn borer^44^. Based on its cDNA sequence from assembled Asian corn borer transcriptome^43^, primers (Table S1) were designed to amplify the full-length cDNA encompassing the entire reading frame using cDNA from the whole body of fifth instar larvae collected 20 h after injection with 3 μl of *M. luteus* (3 μg/μl). The products were cloned into pMD19-T vector, and the nucleotide sequences were confirmed by DNA sequencing. In addition to expected *SP8*, another clip domain serine protease gene with 82.4% identity to *SP8* in nucleotide sequences was obtained during this process. It was designated as *SP105*.

We carried out a series of sequence analysis for identified *SP8* and *SP105*. The deduced amino acid sequences were obtained by using the Translate tool provided by the Swiss Institute Bioinformatics. Analysis of deduced amino acid sequences, including prediction of signal peptide, molecular weight, isoelectric point, and glycosylation sites, was executed in the EXPASY (Expert Protein Analysis System) proteomics server (http://www.expasy.org). Multiple sequence alignment was performed by using the CLUSTALW program, along with the same region in other invertebrate serine proteases with defined functions. The information about the sequences (with GenBank accession number) used for the alignment were found in the figure legend for Fig. S1. Phylogenetic trees were constructed by the neighbor-joining method using MEGA Version 5 software^53^. For neighbor-joining method, gaps were treated as characters, and statistical analysis was performed using the bootstrap method with 1000 replicates.

### Quantitative reverse transcriptase (qRT)-PCR analysis of the expression profiles of *SP105*

To investigate the transcriptional changes of *SP105* during the various stages of Asian corn borer, total RNA samples were individually prepared (*n* = 5) from three different stages including egg, larvae, and pupa using TRNzol Reagent (TIANGEN). One μg of RNA equally from 5 individual RNA samples in each stage was treated with DNase I and converted into first-strand cDNA using FastQuant RT Kit (TIANGEN). The cDNA products independently from 3 biological replicates were diluted 10-fold for use as template in qRT-PCR experiments. Specific primers were designed and listed in Table S1. Asian corn borer ribosomal protein L8 (*rpL8*) was used as an internal standard to mormalize the expression levels. The qRT-PCR was performed on a Applied Biosystems 7500 Real-Time PCR System (Life Technologies) using the SYBR Premix EX Taq^TM^ Kit (TAKARA), according to the manufacturer’s instructions. The thermal cycling conditions for qRT-PCR were 95 °C for 30 s followed by 40 cycles of 95 °C for 30 s and 60 °C for 34 s, then 95 °C for 15 s, 60 °C for 1 min, 95 °C for 30 s, and 60 °C for 15 s. The relative expression of genes was calculated using 2^−ΔΔCt^ method^54^.

To determine the expression patterns of *SP105* in different tissues of Asian corn borer, total RNA samples were isolated separately from combined heads, midguts, fat bodies, and hemocytes from 20 day 0 fifth instar larvae. The synthesis of first-strand cDNA and qRT-PCR analysis was performed as described above.

To check the expression profiles of *SP105* under different inducement conditions, day 1 fifth instar larvae from the same batch were injected into the hemocoel with 3 μl of sterile water containing formaline-killed *E. coli* DH5α (2 × 10^5^ cells/μl), dried *M. luteus* (3 μg/μl), *B. bassiana* suspension (2 × 10^5^ conidia/μl, *B. bassiana* conidia suspension was prepared as described previously^55^), or sterile water as a control. After 20 h (10 h for *B. bassiana* treatment), each five larvae from challenged and control group were collected, and total RNA samples were individually prepared. The following qRT-PCR analysis was conducted as described above.

### Preparation of recombinant Asian corn borer proSP105 and prophenoloxidase-2 (PPO2)

To produce recombinant proSP105, a cDNA fragment encoding the entire proSP105 coding region including the signal peptide was amplified by PCR using primers listed in Table S1 and cDNA from *M. luteus*-injected larvae. The forward primer included a *Bam*H I site, and the reverse primer contained three codons for glycine and six codons for histidine residues followed by a stop codon and a *Not*I site. The PCR product was cloned into pMD19-T vector and then digested with *Bam*H I and *Not*I (TaKaRa). The digested product was recovered by agarose gel electrophoresis, and then inserted into the corresponding restriction sites in pFastBac1 vector (Invitrogen). The resulting proSP105 plasmid, after sequence confirmation, was used as template to produce mutant proSP105 (proSP105_Xa_) plasmid according to Chiu’s method^56^. In proSP105_Xa_, the predicted activation site ADNK^163^ was replaced with IEGR^163^ to allow the cleavage and activation by commercially available bovine Factor Xa^14^. After sequence verification, the resulting plasmids were used to generate a recombinant baculovirus using Cellfectin^®^II Reagent (Invitrogen). For the production of proSP105_Xa_, Sf9 cells (2 × 10^6^ cells/ml) in 500 ml of Insect-Xpress protein-free medium (Lonza) were infected with the recombinant baculovirus at multiplicity of infection (MOI) of 3, then incubated at 28 °C with shaking at 150 rpm. The culture was harvested at 48 h post infection, and cells were removed by centrifugation at 5000 × *g* for 15 min at 4 °C. The cell-free medium was used to further purify recombinant proteins following the method described previously^44^.

For expressing recombinant Asian corn borer prophenoloxidase-2 (OfPPO2), the coding region of mature OfPPO2 was amplified by PCR using specific primers listed in Table S1, in which *Nco*I and *Xho*I sites were added into the 5′-end of forward and reverse primers, respectively. The PCR products were digested and subcloned into the same restriction sites in the expression vector pET28a (Novagen). After sequence verification, the plasmids were used to transform *E. coli* strain BL21 (DE3). The OfPPO2 was then expressed and purified following the methods described previously^46^. Five milligram of purified OfPPO2 was used as antigen for the production of a rabbit polyclonal antiserum (Beijing CoWin Bioscience Co. Ltd.).

### SDS-polyacrylamide gel electrophoresis (SDS-PAGE) and immunoblot analysis

Protein concentrations were determined using Bradford Reagent Solution (Sangon) with bovine serum albumin as a standard. For SDS-PAGE, protein samples were treated with 5× SDS sample buffer containing Dithiothreitol (DTT) at 95 °C for 5 min and then separated by 10% SDS-PAGE (7.5% SDS-PAGE for OfPPO). Proteins were detected by staining with coomassie brilliant blue. For immunoblot analysis, proteins were transferred onto a nitrocellulose membrane and detected with mouse anti-His (1:2,000), or rabbit anti-Asian corn borer PPO2 (1:500) as primary antibodies. Antibody binding was visualized using alkaline phosphate-conjugated goat anti-mouse or anti-rabbit IgG (diluted 1:2,000) and 5-bromo-4-chloro-3-indolyl phosphate/Nitro blue tetrazolium (BCIP/NBT) staining buffer containing 165 μg/ml BCIP and 330 μg/ml NBT in 100 mM Tris (pH 9.5), 150 mM NaCl, and 5 mM MgCl_2_.

### Activation of recombinant proSP105_Xa_ by Factor Xa

To test whether proSP105_Xa_ could be activate by Factor Xa, 0.25 μg of purified recombinant proSP105_Xa_ was incubated with 0.2 μg of bovine Factor Xa (New England Biolabs) in the reaction buffer (20 mM Tris-HCl, pH 8.0, 150 mM NaCl, 2 mM CaCl_2_, pH 8.0) at 37 °C for 4 h. The mixtures were separated by 10% SDS-PAGE followed by immunoblot analysis with anti-His as primary antibodies.

The activation of proSP105_Xa_ was confirmed by measuring the amidase activity of activated SP105_Xa_ with 200 μl of 50 μM acetyl-Ile-Glu-Ala-Arg-*p*-nitroanilide (IEAR*p*NA) in 0.1 M Tris-HCl (pH 8.0), 0.1 M NaCl and 5 mM CaCl_2_ as colorimetric substrate. The amidase activity was measured by monitoring changes in absorbance at 405 nm in a microplate reader (Bio-Tek Instrument, Inc.). One unit of amidase activity was defined as Δ*A*_405_/min = 0.001.

### Assays of Asian corn borer PPO activation by SP105_Xa_

To measure the ability of recombinant SP105 in activating Asian corn borer PPO *in vivo* and *in vitro*, 0.2 μg of proSP105_Xa_ or Factor Xa-activated SP105_Xa_ were incubated at 37 °C with 0.2 μg of purified OfPPO2 or 0.5 μl of plasma collected from day 1 fifth instar Asian corn borer larvae. In control reactions, active SP105_Xa_ was replaced with Factor Xa alone. One hour later, reaction mixtures were subjected to 7.5% SDS-PAGE and immunoblot analysis. To determine PO activity, 0.7 μg of recombinant proSP105_Xa_ or SP105_Xa_ were mixed with 0.5 μl of plasma. Additionally, 1.6 μg of recombinant proSP105_Xa_ or SP105_Xa_ were incubated with 20 μg of purified OfPPO2. After incubation at either room temperature for 10 min (plasma) or 37 °C for 30 min (recombinant OfPPO2), PO activity in the reaction mixtures was measured using dopamine as substrate^13^. One unit of PO activity was defined as the amount of enzyme producing an increase in absorbance (Δ*A*_490_) of 0.001 per min.

### Detection of SDS-stable serpin-3/SP105 complexes by immunoblot analysis

Purified recombinant proSP105_Xa_ (0.2 μg) was activated by Factor Xa as described above, and mixed with purified serpin-3^46^ at molar ratio of 1:1 (proSP105:serpin-3). In control samples, proSP105_Xa_ or Factor Xa was omitted from the mixture. After incubation at room temperature for 30 min, the reaction mixtures were subjected to 10% SDS-PAGE and immunoblot analysis with mouse anti-His (1:2,000) or rabbit anti-serpin3 (1:500) as primary antibodies.

### Analysis of SP105 inhibition by serpin-3 with IEAR*p*NA as substrates

To directly measure the inhibitory potential of serpin-3 on SP105 function, we incubated 0.3 μg of SP105_Xa_ with recombinant serpin-3 at different molar ratios. In control reactions, the same amount of Factor Xa used to activate proSP105_Xa_ was substituted for active SP105_Xa_. After incubation at room temperature for 30 min, the residual amidase activity was measured as described above. Amidase activity of SP105_Xa_ was defined as the activity of SP105_Xa_ minus the activity of Factor Xa alone.

### Analysis of SP105 inhibition by serpin-3 using OfPPO2 as substrates

Activated SP105 (0.2 μg) was mixed with serpin-3 at a molar ratio of 5:1 (serpin-3:SP105). After incubation at room temperature for 30 min, 0.3 μg of OfPPO2 was added to the reaction mixtures, and incubated at 37 °C for 1 h. The cleavage of OfPPO2 in the mixtures was visualized by immunoblot analysis using antiserum against OfPPO2 (1:500 diluted) or His tag (1:2,000 diluted). The residual PO activity in the mixtures was measured using dopamine as substrate as described above.

## Additional Information

**How to cite this article:** Chu, Y. *et al*. Serine protease SP105 activates prophenoloxidase in Asian corn borer melanization, and is regulated by serpin-3. *Sci. Rep.* **7**, 45256; doi: 10.1038/srep45256 (2017).

**Publisher's note:** Springer Nature remains neutral with regard to jurisdictional claims in published maps and institutional affiliations.

## Supplementary Material

Supplementary Information
