# Supplementary material for: Serine protease SP105 activates prophenoloxidase in Asian corn borer melanization, and is regulated by serpin-3
Source: Sci Rep. 2017 Mar 30;7:45256. doi: 10.1038/srep45256 (PMC5372168; doi:10.1038/srep45256)
Supplement: Supplementary Information [file srep45256-s1.pdf]

Serine protease SP105 activates prophenoloxidase in Asian corn borer melanization, and is regulated by serpin-3

Yuan Chu, Fang Hong, Qizhi Liu, Chunju An*

Department of Entomology, China Agricultural University, Beijing, China, 100193

*Correspondence and requests for materials should be addressed to C.A. (email: anchunju@cau.edu.cn)

Supporting information

The following supplementary material is available:

Table S1 Oligonucleotides used for cloning, RT-PCR, and plasmid construction.

Figure S1 Phylogenetic analysis of the catalytic domain of Asian corn borer SP8 and SP105 and other insect clip domain serine proteases with defined function. The used amino acid sequences included *O. furnacalis* SP1, SP13, SP8 (KT751522) and SP105 (KT751521); *A. gambiae* CLIPB9 (HM070255); *B. mori* BAEEase (ABB58762); PPAE (NP_001036832); *D. melanogaster* Easter (NP_524362), Grass (NP_733197), Hayan (CG6361); MP1 (CG1102), MP2 (CG3066), Persephone (NP_573297), SPE (NP_651168), Spirit (NP_727276), Snake (NP_524338); *H. diomphalia* PPAF1 (BAA34642); *M. sexta* HP6 (AY627782), HP8 (AY627784), HP21 (AY627797), PAP1 (AY789465), PAP2 (AY077643), PAP3 (AY188445); *T. molitor* SAE (AB363979), SPE (AB363980). Numbers at the branches indicate bootstrap value, as a percent of 1000 repetitions. The # of clip domain means the number of clip domain in the deduced amino acid sequences, predicted based on Jiang's definitions.

Figure S2 Detection of activation of proSP8$_{Xa}$ by bovine Factor Xa. Twenty μl of conditioned medium containing recombinant proSP8$_{Xa}$ was incubated with 0.6 μg of Factor Xa at 37 °C overnight. The mixtures were separated by 10 % SDS-PAGE followed by immunoblot analysis with anti-His as primary antibodies. The sizes and positions of the molecular weight standards are indicated on the *right*.

Figure S3 Activation assay of wild type proSP105 exposure to plasma proteins or mutated proSP105$_{Xa}$ incubated with Factor Xa. Twenty μl of conditioned medium containing wild type proSP105 or mutated proSP105$_{Xa}$ was incubated with 0.5 μl of Asian corn borer plasma or 0.2 μg of Factor Xa, respectively at 37 °C. After 1 h, the mixtures were subjected to 10 % SDS-PAGE followed by immunoblot analysis using His antiserum. The sizes and positions of the molecular weight standards are indicated on the *right*. Circle, proSP105 or proSP105$_{Xa}$; asterisk, catalytic domain of proSP105 or proSP105$_{Xa}$.

Figure S4 Cleavage analysis of purified recombinant proSP105$_{Xa}$ and proSP1$_{Xa}$ by plasma proteins. Purified recombinant proSP105$_{Xa}$ (0.2 μg) or proSP1$_{Xa}$ (0.4 μg) was mixed with Factor Xa (0.2 μg) or collected Asian corn borer plasma (0.5 μl, 1:10

diluted) at 37 °C. After 1 h (10 min for plasma), the reaction mixtures were subjected to immunoblot assay using anti-His as primary antibodies. The sizes and positions of the molecular weight standards are indicated on the *right*. Circle, proSP105$_{Xa}$; asterisk, catalytic domain of proSP105$_{Xa}$; square, proSP1$_{Xa}$; star, catalytic domain of proSP1$_{Xa}$.

Figure S5 Cleaving analysis of *D. menalogaster* PPO1 by Factor Xa-activated SP105$_{Xa}$. Purified proSP105$_{Xa}$ (0.2 µg) was activated with 0.2 µg of Factor Xa, and then incubated with 0.3 µg of purified recombinant *D. menalogaster* PPO1 (DmPPO) at 37 °C for 1 h. The reaction mixtures were subjected to immunoblot assay using His antiserum. The sizes and positions of the molecular weight standards are indicated on the *right*. Hollow diamond, PPO1 zymogen; solid diamond, cleaved and activated PO1.

Figure S6 SP1 failed to cleave wild type proSP105. Purified recombinant proSP1$_{Xa}$ (0.4 µg) was activated by 0.2 µg of Factor Xa, and mixed with 20 µl of conditioned medium containing wild type proSP105 at 37 °C for 1 h. The reaction mixtures were subjected to immunoblot assay using His antiserum. The sizes and positions of the molecular weight standards are indicated on the *right*. Circle, proSP105$_{Xa}$; square, proSP1$_{Xa}$; star, catalytic domain of proSP1$_{Xa}$.

Table S1 Oligonucleotides used for RT-PCR and plasmid construction

	**Forward primers**	**Reverse primers**
Cloning of SP8 based on transcriptome sequence:		
	5’-CATAGCTCCGCCGGCCGTC-3’	5’-TGAAGTATTTCAAGG-3’
Amplification of ORF of proSP8 and proSP105:		
SP8	5’-CTTAGATAAAAATGAAGC-3’	5’-TCAAGGCTGAATATTGCC-3’
SP105	5’-CATAGCTCCGCCGGCCGTC -3’	5’-AGTATTTCAAGGGTAAATGTTTTCCC-3’
qRT-PCR:		
SP105	5’-GACTCAAAGCAGATTTATG-3’	5’-GACTACTCCAAGCATATC-3’
rpL8	5’-AAGCGAGGAACATCAGCC-3’	5’-GGTCTTGCCACCACGAAT-3’
Amplification of full-length proSP105, including signal peptide with restriction sites for cloning in pFastBac1:		
	5’-GCGGATCCCTTAGATAAAAATGAAGC-3’	5’-ATG*ACCTCCTCC*GAGGGTAAATGTTTTCCC-3’
		5’-ATGCGGCCGCTTAATGGTGATGGTGATGATG *ACCTCCTCC*-3’
	Added *BamH*I site is underlined.	Codon for three extra glysine is italicized.<br>Added *Not*I site underlined.<br>The reverse complement sequence for 6 histidine residues inserted in front of the stop codon is double-underlined.
SP105 mutagenesis:		
	SP105FT: 5’-GGCGGAATAGAAGGTCGGATCACAGGGGGTTCTGCAAC-3’	
	SP105FS: 5’-TCACAGGGGGTTCTGCAAC3’	
	SP105RT: 5’-TGTGATCCGACCTTCTATTCCGCCGTCGAGGCCGCAGCACT-3’	
	SP105RS: 5’-TCCGCCGTCGAGGCCGCAGCACT-3’	
	sequence encoding IEGR instead of wild-type ADNK is underlined.	
Amplification of full-length PPO2 for cloning in pET28a:		
	5’-CC***ATG***GGCGCGGACGTTGTGAAAAGCT-3’	5’-CTCGAGGCCCTGAGTGGGGTTCCTGGGGT-3’
	Added *Nco*I site is underlined.<br>Codon for start codon is bold italicized.	Added *Xho*I site is underlined.

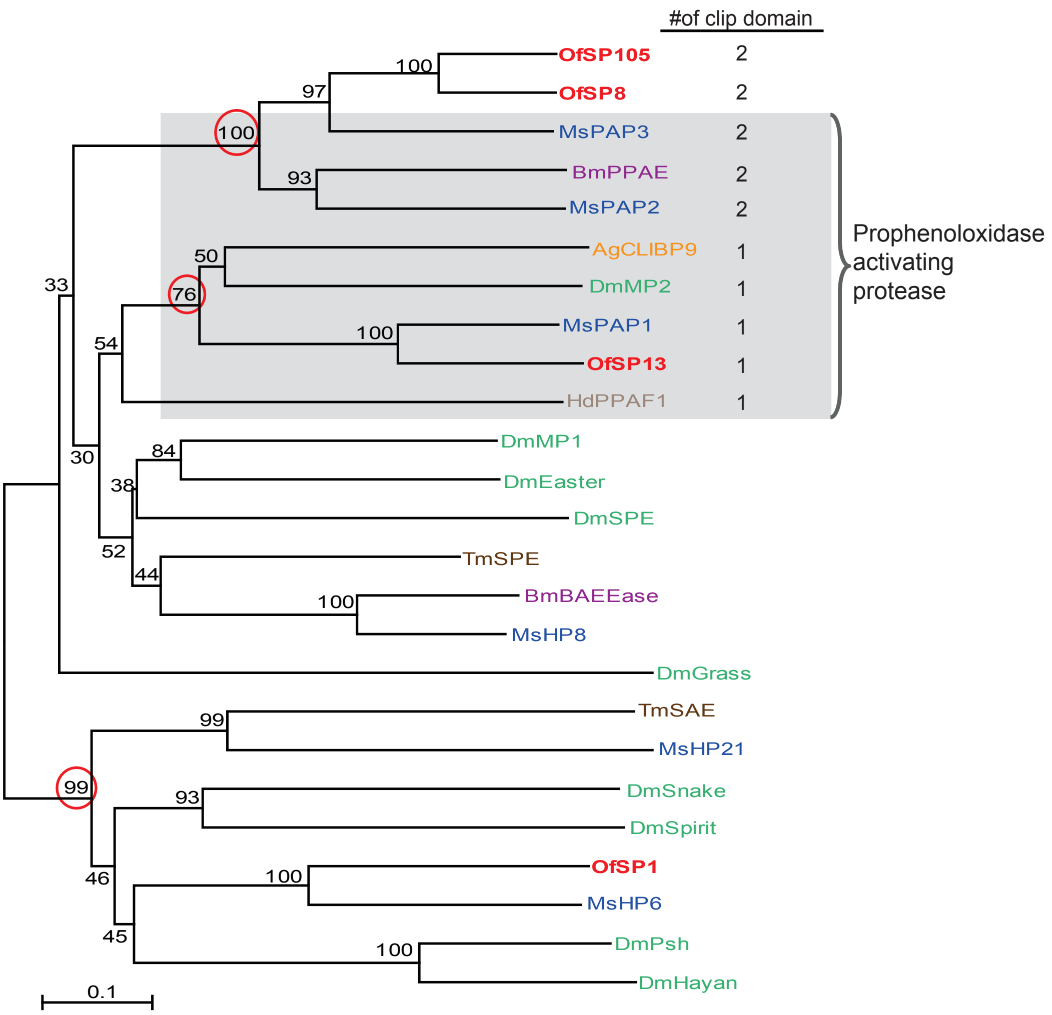
#of clip domain
OfSP105 2
OfSP8 2
MsPAP3 2
BmPPAE 2
MsPAP2 2
AgCLIBP9 1
DmMP2 1
MsPAP1 1
OfSP13 1
HdPPAF1 1
Prophenoloxidase activating protease
DmMP1
DmEaster
DmSPE
TmSPE
BmBAEEase
MsHP8
DmGrass
TmSAE
MsHP21
DmSnake
DmSpirit
OfSP1
MsHP6
DmPsh
DmHayan
100
97
100
93
50
76
100
54
33
30
84
38
52
44
100
99
99
93
46
100
45
100
0.1

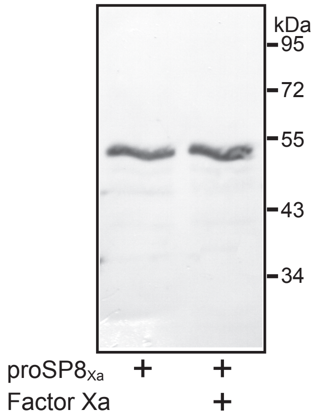
kDa
95
72
55
43
34
proSP8Xa
+
+
Factor Xa
+

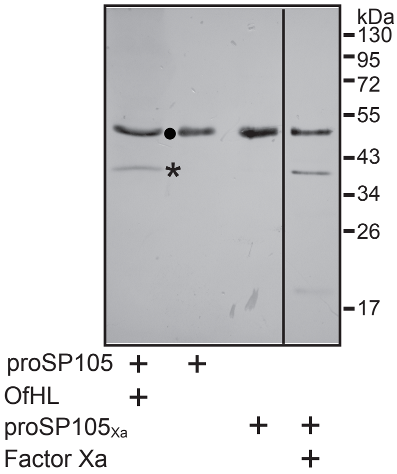
kDa
130
95
72
55
43
34
26
17
*
proSP105 + +
OfHL +
proSP105Xa + +
Factor Xa +

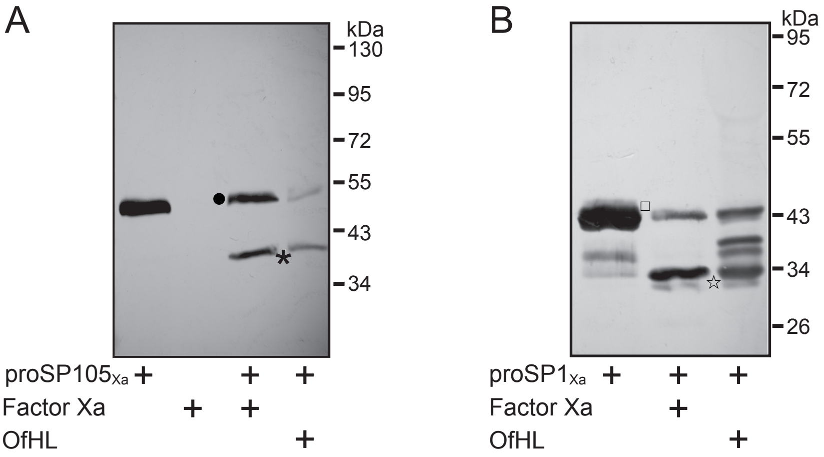

A
kDa
130
95
72
55
43
34
proSP105Xa + + +
Factor Xa + +
OfHL +
B
kDa
95
72
55
43
34
26
proSP1Xa + + +
Factor Xa +
OfHL +

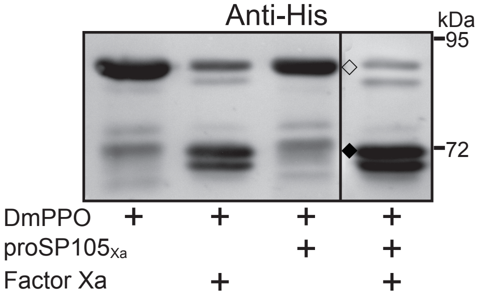
Anti-His
kDa
95
72
DmPPO + + + +
proSP105Xa + +
Factor Xa + +

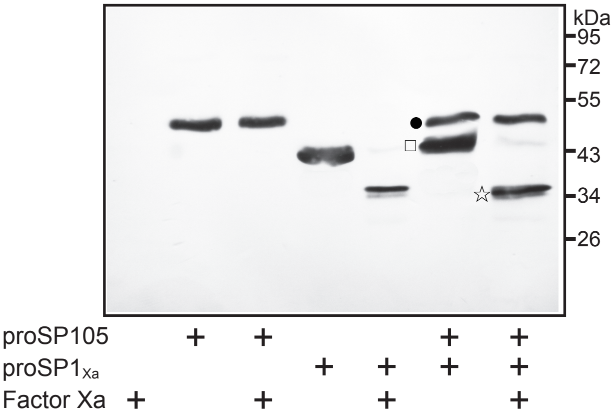
kDa
95
72
55
43
34
26
proSP105 + + + +
proSP1Xa + + + +
Factor Xa + + + +
